# Filler Effect in Shotcrete

**DOI:** 10.3390/ma12193221

**Published:** 2019-10-01

**Authors:** Isabel Galan, Lukas Briendl, Maria Thumann, Florian Steindl, Rudolf Röck, Wolfgang Kusterle, Florian Mittermayr

**Affiliations:** 1 Institute of Applied Geosciences, Graz University of Technology, 8010 Graz, Austria; 2 Institute of Technology and Testing of Building Materials, Graz University of Technology, 8010 Graz, Austria; 3 Faculty of Civil Engineering, Ostbayerische Technische Hochschule Regensburg, 93025 Regensburg, Germany; 4 Department of Structural Engineering and Materials Sciences, University of Innsbruck, 6020 Innsbruck, Austria

**Keywords:** acceleration, hydration, mechanical properties, CaCO_3_

## Abstract

The effects of fine limestone powder on the early hydration of cementitious systems accelerated by means of alkali-free aluminum sulfate based products, commonly used for shotcrete applications, were investigated in the course of laboratory and real scale tests. In binary (CEM I + limestone) and ternary (CEM I + limestone + slag) systems the addition of fine limestone led to an enhancement of the hydration degree and strength development at early times (<24 h). The formation of ettringite, aluminate hydrates, and C–S–H is affected by the joint action of the setting accelerator and the fine limestone. Accelerator and limestone, in combination with the cement, can be optimized to enhance ettringite and silicate reaction, in some cases coupled with aluminate reaction inhibition, to produce mixes suitable for sprayed concrete applications. Such optimization can help to reduce the cement content in the mixes without compromising the early strength development of the shotcrete.

## 1. Introduction

### 1.1. Acceleration of Shotcrete

Hydration reactions of cementitious systems can be accelerated by different means and for various purposes. In particular, the type of admixtures known as ‘setting accelerators’ enhance the very early hydration of wet-mix shotcrete (or sprayed concrete) [1]. In this case, the wet-mix, consisting of cement with or without supplementary cementitious materials (SCMs), aggregates, water, and admixtures, such as superplasticizer and retarder, is pumped to the spraying nozzle where it is mixed with the accelerator and compressed air while being shot. The fast setting, achieved thanks to the accelerator, together with the application (shooting) of the concrete, is crucial to ensure proper adhesion to the surface where it is applied and adequate mechanical performance during the first minutes to hours. These special requirements are mandatory in underground construction works like tunneling, where immediate rock support after blasting is provided by the shotcrete. Currently, the setting accelerators most commonly used for shotcrete are alkali-free (or with very low alkali content) and based on aluminum sulfate (or aluminum hydroxysulfate). These admixtures, either in the form of solutions or suspensions, lead to the instantaneous massive formation of ettringite right after contacting the wet mix [2]. The ettringite network formed constitutes the initial hardened matrix in which the rest of the hydrated phases forms and develops at later stages. In these accelerated systems, earlier dissolution of alite has been reported to be favored by the higher consumption of calcium ions during the formation of ettringite [3] and also by the acid contained in the accelerator [4], thus moving the onset of the silicate reaction forward. The early ettringite formed has even been hypothesized to act as nucleation seed for the formation of C–S–H [5]. On the other side, massive formation of ettringite on the cement particles may also act as a barrier, inhibiting calcium silicate hydrate formation [3,4,6]. Together with the accelerator composition and dosage and the cement mineralogy, the amount and type of setting regulators control the hydration sequence and the development of mechanical properties [7,8,9,10].

### 1.2. Effects of Fine Limestone Fillers in Concrete

The substitution of cement by SCMs and fillers [11] has become a common practice in the last decades in many concrete applications [12]. Reduction of CO_2_ emissions and improvement of durability properties are among the most important purposes of using these materials. In particular, the incorporation of fine fillers to enhance hydration and increase concrete strength has been a research topic for the last 20 years [13,14,15,16,17,18,19,20,21,22,23,24,25,26,27,28,29,30,31]. Despite the numerous investigations, the “filler effect” is still a controversial issue, and the mechanisms by which fine limestone works better than other fillers are not totally agreed upon. In the presence of fine limestone, formation of hemi- and monocarboaluminate (Hc and Mc, respectively) is favored over monosulfoaluminate (Ms), in turn indirectly stabilizing ettringite [13]. This occurs also in the presence of fine dolomite, where additionally hydrotalcite and brucite may form [14]. The stabilization and formation of ettringite from the sulfate liberated during carbonation and formation of CO_3_-AFm phases (e.g., Hc and Mc) leads to an increase in the molar volume of hydrates, with a consequent space-filling and reduction of porosity and permeability [24,25], and thus an increase in strength [26]; this latter effect is however only true for low quantities of calcite replacement. According to Zajac et al. [27], calcite starts to react once gypsum is depleted, the dissolution rate of calcite being controlled by the formation of CO_3_-AFm (Hc and Mc), which in turn is controlled by the availability of aluminum.

Of the several hypotheses proposed to explain the hydration enhancement in mixes containing fine fillers, the provision of extra nucleation sites seems to be the one with the widest acceptance [26,28,29]. The filler does not have to be reactive to provide extra nucleation sites [28]; however, the acceleration effect of quartz is considerably lower than that of limestone [29,30], and so is the effect of dolomite [14] and aragonite [31] as compared to calcite. Another possible explanation for the enhancement of hydration is the reduction of the thickness of the hydrated layer around cement grains in the presence of fine fillers, facilitating further hydration of the anhydrous part [28]. The water in the system plays an important role in the accelerating effect of fine fillers: Lower water/solid ratios (0.35) promote acceleration of paste hydration to a much greater extent than higher ratios (0.435), where the effect may even be negligible [16]. Similarly, decreasing the grain size of limestone filler [15,17], or, more precisely, increasing the surface ratio between filler and cement [29] proportionally enhances the rates of hydration reactions. Related to the provision of nucleation sites, Oey et al. [29] hypothesized that the energy barrier for C–S–H nucleation is lower for calcite than for quartz. Additionally, to compensate the unbalanced charge which follows from the sorption of CO_3_^2−^ ions on the C–S–H, hydroxide ions would be released, thus elevating the pH value and promoting further hydrate growth. Lower oversaturation with respect to C–S–H and higher undersaturation with respect to C_3_S are the driving forces for the enhancement of the silicate reaction in the presence of fine calcite [18]. The interparticle distance and the consequent shearing conditions are also proposed as important parameters in the filler effect [19]: Similar enhanced hydration is observed in mixes where shearing is increased by the presence of limestone (or another filler) and in those where a higher mixing speed is applied. According to Ouyang [20], the interaction between the filler and the calcium ions determines the adhesion between C–S–H and filler: In the case of calcite the acid-base interactions between the filler and the Ca^2+^ ions lead to a strong bond (most likely ionic-covalent), whereas in the case of silica the adhesion is governed by an attractive ion–ion correlation force.

In multiple systems, including cement and SCMs, fly ash, slag and metakaolin contribute to the formation of additional calcium aluminate hydrates. The aluminates liberated during the pozzolanic reaction decrease the sulfate/aluminate ratio, promoting ettringite decomposition and formation of Ms. In the presence of calcite, however, ettringite is stabilized and, through reaction with the extra aluminates available, CO_3_-AFm forms [21,22]. According to Adu-Amankwah et al. [23], slag hydration is accelerated in the presence of fine limestone due to the lowering of aluminum concentration in the solution (because of its incorporation in ettringite and CO_3_-AFm) and the extra space available for hydrate growth.

### 1.3. Effects of Fine Limestone Fillers in Wet-Mix Shotcrete

The replacement of cement by SCMs and fillers in shotcrete is, in practice, restricted to very few combinations, mainly due to lack of experience and national and international regulations. A combination of granulated blast furnace slag (GBFS), fly ash and limestone in different proportions, but not exceeding 35% cement replacement, is commonly used in tunnels in the Austrian Alps region (OENORM B 3309-1). This formula has proven to help reducing the sintering potential in tunnel drainage systems [32]. In Australia typical wet-mix shotcrete mixes used for mining and tunneling projects include 20–40 kg/m^3^ silica fume and up to 60 kg/m^3^ fly ash [33]. Despite SCMs being mentioned in various national guidelines for shotcrete [34,35,36] their use is still not very common and official recommendations for specific combinations are scarce.

The hydration progress of shotcrete in the presence of fine fillers has not been fully explored yet despite its importance. Very few authors have dealt with the so-called filler effect in accelerated systems like shotcrete. Stefanoni et al. [37] measured higher mechanical strength in accelerated mortars made with calcium carbonate based aggregates compared to those made with quartz aggregates. The authors state that limestone aggregates improve the performance of alkali-free aluminum sulfate-based accelerators, favoring the formation of ettringite. Salvador et al. [9] also reported hydration enhancement in accelerated mixes with limestone (and gypsum), attributing the effect to the avoidance of accelerated undersulfated reactions of tricalciumaluminate through formation of carbonate-AFm phases. The dissolution of limestone is promoted by the low pH of the setting accelerator, which then promotes aluminate reaction; in the presence of hemihydrate instead of gypsum, due to the higher solubility of hemihydrate and the common ion (Ca^2+^) effect, Salvador et al. reported an inhibition of limestone dissolution and thus a limited efficiency of the filler. Additionally, limestone particles may act as nucleation sites for the precipitation of aluminate hydrates [9].

Sprayed concrete systems are much more complex than ‘normal’ concrete mixes and the deconvolution of the various acceleration processes that take place during hydration has proven much more challenging. The present work aims at shedding light on these accelerated systems, in the presence and absence of fine limestone (FLS) by analyzing how the filler affects the formation of ettringite, aluminate hydrates and C–S–H, and how the synergistic effect of the setting accelerator and the filler modifies the hydration progress. GBFS was included in some of the systems: (i) To further prove the filler effect in low cement matrices, (ii) to study its possible influence in the early hydration process, and (iii) to analyze the effect of increasing the FLS content while keeping the cement content constant in the binder. Mixes were sprayed in the lab and compared to non-accelerated mixes by means of isothermal calorimetry, shear modulus determination, X-ray diffraction (XRD), Fourier-transform infrared spectroscopy (FTIR), thermogravimetry (TG) and scanning electron microscopy (SEM). Real scale spraying tests were also carried out with selected mixes to corroborate the effects observed in the lab.

## 2. Experimental

### 2.1. Materials

For the experiments conducted in the lab two types of cements were used: A CEM I 52.5R (SpC) and a CEM I 52.5N SR0, with a Blaine surface area of 5200 and 5000 cm^2^/g, and a d_50_ of 7.1 and 8.0 μm, respectively. The composition of the cements is shown in Table 1 and Table 2. The cement CEM I 52.5R (SpC) is considered suitable for sprayed concrete; the CEM I 52.5N SR0 is a standard cement used for normal concrete exposed to sulfate loaded environments. Noteworthy in the CEM I 52.5N SR0 cement are (i) the high calcite content, which would actually make the cement classify as CEM II, according to EN 197, and (ii) the C_3_A content, ~1.7%. From now on in the paper, CEM I 52.5R (SpC) and CEM I 52.5N SR0 will be called CEM I and CEM SR, respectively. A very fine limestone (FLS) made of 96% calcite, 4% dolomite and traces of quartz, with a d_50_ of 1.2 μm, was chosen as microfiller. For the aggregates a limestone powder (Nekafill) with grain sizes smaller than 125 μm, composed of 86% calcite and 12% dolomite was used. Finally, a GBFS with d_50_ 8.6 μm was selected for some of the mixes. The oxide composition of all materials is given in Table 2.

The chemical admixtures used consisted of a (long-chain) polycarboxylate ether based superplasticizer (SP) (Viscocrete SC 600, Sika, Zürich, Switzerland), a citric acid based retarder (Sikatard 930, Sika, Zürich, Switzerland) and an alkali-free aluminum sulfate based suspension accelerator (Sigunit L5601AF, Sika, Zürich, Switzerland) with a molar ratio Al/S ~0.7). The retarder was added to allow for the lab-scale spraying of the CEM I mixes: This cement proved to be very reactive and mixes without retarder led to tube blockages. Despite this not being the case for CEM SR mixes, retarder was also used to keep mixes comparable, reducing the parameters changed in the various systems.

For the real scale tests, where some of the lab mixes were also sprayed, similar materials were used, except for the aggregates, which were 0–4 and 4–8 mm dolomitic aggregates, and the absence of retarder.

### 2.2. Mixes and Spraying

**Figure 1:**
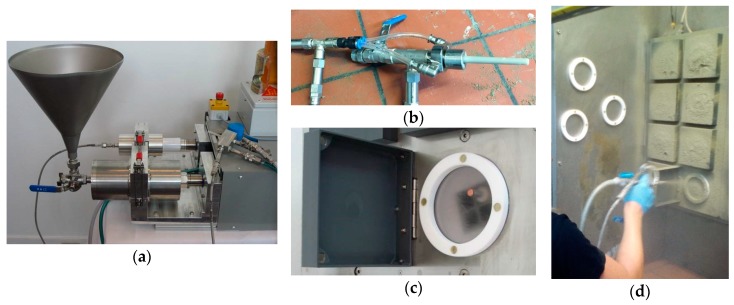
MiniShot spraying device: (**a**) Mix funnel and accelerator pump, (**b**) spraying nozzle with accelerator, cement mix and compressed air connections, (**c**,**d**) spraying cells.

Sprayed and non-sprayed samples were prepared in the lab. The non-sprayed ones consisted of water, cement, FLS filler, GBFS, and limestone powder aggregates. The sprayed samples also included superplasticizer, retarder, and setting accelerator. For both types of samples a Hobart mixer was used to mix all the components except for the accelerator in the sprayed samples, which was added at the nozzle of the spraying device called “MiniShot” [38,39] (Figure 1), together with the compressed air. The MiniShot spraying device used allows for the lab-scale spraying of ‘micro-concrete’ or ‘fines-based concrete’, that is, to prove the pumpability and sprayability of the concrete mix at the corresponding scale. The mixing sequence was as follows: (1) The binder components (cement, GBFS, and FLS), and in most cases the Nekafill aggregates, were mixed with the water in the Hobart mixer for 2.5 min, (2) the mix was left to rest for 10 min, (3) superplasticizer and retarder were added and mixed for 1.5 min, (4) the mix was left to rest for about 2 min before starting the spraying process, and (5) the mix was then sprayed together with the accelerator and the compressed air. The times used for the mixing, in particular the 10 min delay for the SP addition, were found to produce an optimal system performance.

Table 3 summarizes the mixes that were prepared. As stated, all these mixes were: (i) Mixed in Hobart (with no chemical admixtures), and (ii) sprayed with MiniShot device (with the three admixtures). The mass percentages included in Table 3 refer to the binder composition (including cement, GBFS, and FLS). A sample with each type of cement and no aggregates was mixed and sprayed with the aim of comparing both cements without the possible influence of the fine aggregates (mixes one and eight). From the 10 mixes including aggregates, four of them were selected to be tested in real scale spraying trials. It is worth noting that in mixes three, four, 10 and 11 a certain weight percent of cement was substituted by FLS, whereas in mixes six and seven the FLS substituted the GBFS (calculated by mass), keeping the cement content constant.

The exact composition of the mixes sprayed in the lab is given in Table 4. The water/binder (w/b) ratio used was optimized for both types of cement and kept constant for all mixes: The higher water demand of the CEM I resulted in slightly higher w/b ratios than in the CEM SR mixes. Percentages of SP, retarder, and accelerator are reported with respect to the binder mass (cement + GBFS + FLS). The quantities of SP added were adjusted to achieve adequate workability, similar for all mixes. The accelerator dosage was also kept constant at 6 g per 100 g binder. Finally, the retarder content varied between 0.3 wt.% in samples with low clinker content, and 0.5 wt.% in those with high clinker content. It is important to remark that the replacement of cement by FLS in the GBFS-free samples resulted in an increase of the w/c and accelerator/cement (acc/c) ratios. In the GBFS-containing samples the cement content and, consequently, the w/c and acc/c ratios were kept constant. The mass ratio aggregate (<125 μm)/binder was kept between 0.31 and 0.34 in all cases. The temperature in the MiniShot lab was kept constant at 20 °C.

**Figure 2:**
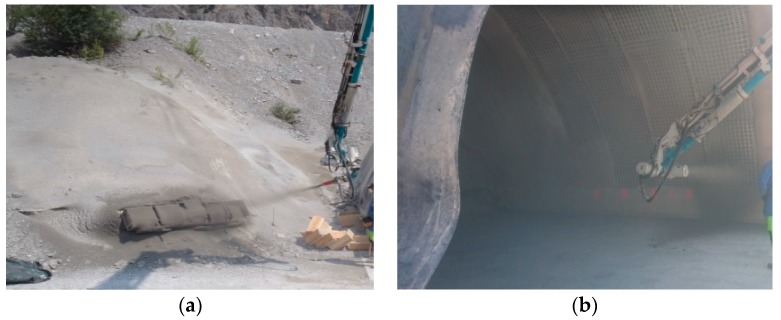
Real scale spraying test (**a**) on the panels and (**b**) on the tunnel walls, from [40].

**Figure A1:**
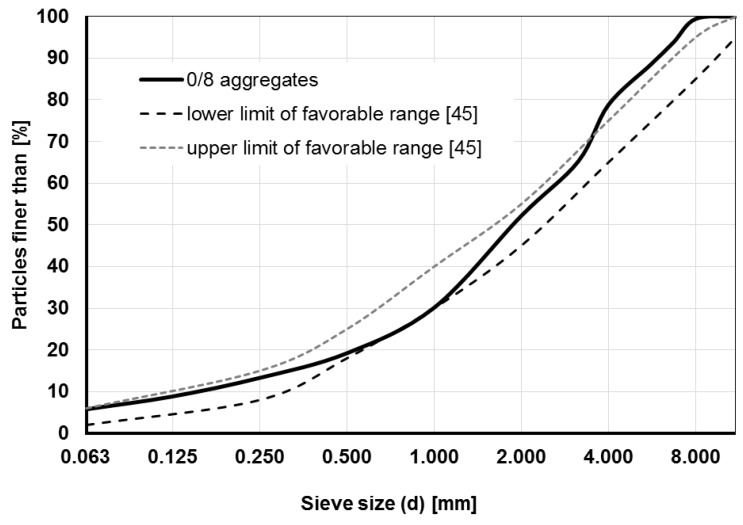
Grain size distribution of the aggregates used for the real scale tests, plotted together with the curves from the Austrian Guidelines for Shotcrete [45].

The composition of the mixes sprayed in real scale is given in Table 5. From the total mass of aggregates, 75% correspond to the 0–4 mm and 25% to the 4–8 mm. The grain size distribution of the aggregates is provided in the Appendix A (Figure A1). The mass ratio aggregate/binder was kept between 4.5 and 4.7 and the total binder mass between 401 and 411 kg/m^3^. The equipment used for the real scale spraying (CIFA-Hittmayr) had both the pump and the manipulator mounted on a mixer truck. Fresh concrete was mixed at the on-site mixer (~3 m^3^) as follows: Aggregates, binder components, and water were added to the mixer and mixed for about 1 min, after which SP was added, continuing then the mixing for another 90 s. The mixer truck reached the spraying site 5 min later and the spraying started in the next 5–25 min. The spraying throughput was set at 20 m^3^/h and a 17 m^3^ compressor was used for the addition of the compressed air at the nozzle. 3 m^3^ of concrete were sprayed per mix, partially into spraying panels and partially on a test tunnel wall (Figure 2). The distance from the nozzle to the receiving surface was 1.5–2 m. The ambient temperature varied between 15 °C (at night) and 35 °C (during the day). The spraying took place during the day, at 20–30 °C. To ensure proper curing, water was periodically sprinkled over the samples sprayed on the panels.

### 2.3. Methods

Materials’ fineness was measured by means of (i) laser diffraction with dry dispersion (Helos H2395 & Rodos), for the determination of the d_50_, and (ii) Blaine air permeability tests, according to EN 196-6. Oxide composition of the materials was determined by (glass pellets) X-ray fluorescence analysis (XRF), performed on a PW 2404 spectrometer (Malvern Panalytical, Almelo, The Netherlands).

The MiniShot equipment (Figure 1, Sika, Zurich, Switzerland) allowed for the continuous recording of the shear modulus evolution of the shotcrete by means of ultrasound wave amplitude attenuation measurements [41]. Isothermal calorimetry measurements (I-Cal 8000 Calmetrix, Boston, MA, USA) were performed on sprayed and non-sprayed samples at 23 °C. Since the risk of producing inhomogeneous systems is higher in accelerated systems some mixes were selected to repeat the whole mixing and spraying process plus the subsequent shear modulus and isothermal calorimetry measurements. Similar curves were obtained in all cases proving the repeatability of the mixing/spraying/measuring procedure.

Just after spraying and after 3, 6 and 24 h of humid curing, hydration of the samples was stopped by means of solvent exchange (with isopropanol, solid:liquid ratio of ~1:10) followed by drying at 35% relative humidity in inert atmosphere for 1–4 days, which allowed for the removal of the ‘free’ water. These samples were then characterized by means of (i) thermogravimetry (TG), (ii) X-ray diffraction (XRD), (iii) Fourier-transform infrared spectroscopy (FTIR) and (iv) scanning electron microscopy (SEM). TG measurements were performed on a TG 209 F1 Libra (Netzsch, Selb, Germany) with heating from 25 to 1000 °C, heating rate 20 °C/min, and under 10 ml/min N_2_ flux. The data obtained were used to quantify portlandite content and total bound water. To identify the crystalline phases formed in the samples XRD measurements were carried out on a Panalytical X’Pert Pro diffractometer (Malvern Panalytical, Almelo, The Netherlands), with Cobalt radiation, 40 kV and 40 mA, 7°–80°, step size 0.017, and scan speed 0.021°/s. FTIR spectra were collected on a PerkinElmer Frontier spectrometer (PerkinElmer, Waltham, MA, USA) to gain further knowledge of the hydration processes taking place in the different systems. SEM images, used for morphological analysis, were obtained on a DSM 982 Gemini equipment (Zeiss, Oberkochen, Germany), with gold-palladium coating, 5 kV acceleration voltage and 4–5 mm working distance.

For the quantification of the phases by means of the Rietveld refinement method, the HighScore Plus software (Malvern Panalytical, Almelo, The Netherlands) was used. The amorphous content in the samples was calculated by means of the G-factor method [42] with the use of an external standard (NIST, SRM-676a) [43]. For the phase quantification of the anhydrous cements selective dissolution was performed: (i) Salicylic acid-methanol method (SAM) for the dissolution of the silicates and (ii) potassium hydroxide-sucrose method (KOSH) for the dissolution of the aluminates, ferrite, and sulfates [44]. To ease comparison calorimetry data are expressed per g cement, and TG and XRD-Rietveld data in weight percentages referred to the cement weight in the samples (and not to the total sample weight). For this, the proportions of the solid components in the dry mixes (Table 4), together with the water content deduced from the TG measurements were used. In the case of the accelerator, 35% of the total mass was considered solid, according to the data provided by the producer.

The early strength of the real scale shotcrete was measured on-site according to EN 14488-2: from 0.2 to 1 MPa by means of a penetration needle, and from 2 to 16 MPa with a DX 450-SCT powder-activated testing device (Hilti, Schaan, Liechtenstein) equipped with threaded studs [45]. The penetration resistance of the needle and the ratio between the pull-out force of the studs and their penetration depth were converted to compressive strength values with the use of calibration curves.

## 3. Results

### 3.1. Heat of Hydration and Shear Modulus

**Figure 3:**
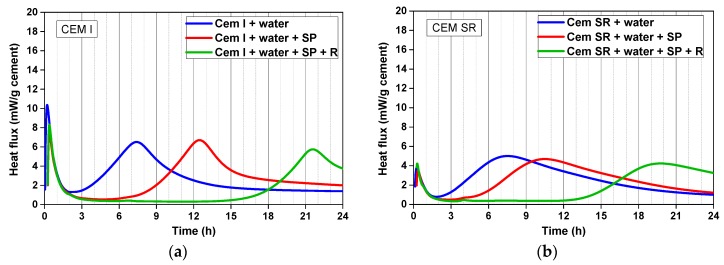
Isothermal calorimetry curves of (**a**) mix one, with CEM I, and (**b**) mix eight, with CEM SR (Table 3 and Table 4), both without aggregates, before accelerator addition.

For the initial characterization of the two cements and the corresponding mixes, heat evolution curves were recorded after (i) mixing the cement with water, (ii) adding the superplasticizer, and (iii) adding the retarder (Figure 3). The first peak of the isothermal calorimetry curves is considerably higher for the CEM I than for the CEM SR mixes with low C_3_A content. However, because the samples were mixed externally and the first minutes between the sample being ready and the measurement start are not included in the graphs, the heights of the first peaks cannot be taken as absolute values. The second peak reaches in both systems its maximum value at the same time, after ~7.5 h, but the peak shape is different (more ‘pointy’ in CEM I) and so are the maximum heat values reached (6.5 and 5.0 mW/g for CEM I and CEM SR, respectively). The addition of the superplasticizer led to a retardation of the second peak in both systems, the effect being in this case more pronounced in the CEM I mixes (with 0.7% SP) than in CEM SR systems (with 0.3% SP). The retarder action (both systems with 0.5% retarder) is similar in both cases, shifting the maximum of the second peak for ~9 h.

**Figure 4:**
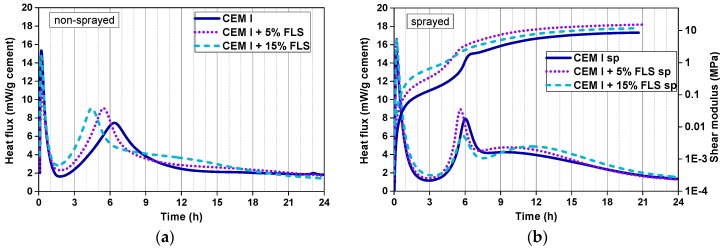
(**a**) Calorimetry curves of non-sprayed samples of mixes two, three, and four (Table 3 and Table 4), with CEM I + FLS; (**b**) calorimetry and shear modulus curves of sprayed samples of mixes two, three, and four (Table 3 and Table 4), with CEM I + FLS.

The effect of substituting cement with the FLS microfiller is very clear in non-sprayed CEM I samples (Figure 4a), where the maximum value of the second peak is reached ~1 and ~2 h earlier in samples with 5% and 15% FLS, respectively. In fact, the impact of the fine calcite aggregates can already be observed in CEM I samples without FLS: The second peak maximum is reached 1 h earlier in systems with aggregates (Figure 4a) than in paste systems (Figure 3a). None of these samples contain SP, retarder or accelerator, and they all have the same w/b ratio, 0.47.

In the lab-sprayed CEM I samples the first noticeable observation is the almost complete disappearance of the retarding effects of retarder (in all mixes 0.5% referred to the cement) and superplasticizer (1.0–1.4% referred to the cement) due to the addition of the accelerator (Figure 4b): The main hydration events take place at similar times as in mixes with no admixtures (Figure 4a). Second, the differences between non-sprayed and sprayed samples are remarkable: (i) Important narrowing of the second peak in the sprayed samples, and (ii) appearance of a pronounced third maximum, only slightly noticeable in the non-sprayed 15% FLS sample and indistinguishable in the 0% and 5% FLS ones. The shift in the second peak due to the FLS is less pronounced in sprayed than in non-sprayed samples, and it does not increase with the FLS content (Figure 4b). The maximum of the second peak in the 5% FLS sample is reached 30 min earlier and the values are higher than in the sample with no FLS. From 5% to 15% FLS, however, the peak does not get shifted to earlier times and the maximum value reached is considerably lower. In this set of samples the accelerator/binder ratio was kept constant (the acc/c ratio increasing with the FLS substitution).

There are certain relations between the evolution of the shear modulus and the heat flux: (i) The initial calorimetry peak corresponds to the initial steep increase of the modulus (1^st^ h), (ii) during the induction period (low heat flux) the modulus continues to grow at a slower pace (~1^st^–5^th^ h), (iii) the highest slope increase of the modulus coincides in time with the maximum heat released during the second peak (~5^th^–7^th^ h), and (iv) after that, the modulus continues growing at a moderate rate during the third hydration stage (up to the 24^th^ h) (Figure 4b). It is important to note that the logarithmic plot helps to visualize the increas of the shear modulus at earlier times. However, it makes the visualization of the ‘later’ events difficult. In fact, after the second peak, the modulus presents an almost linear increase with time up to the third maximum, after which it continues growing at a slower pace during the deceleration period. During the first 4 h higher FLS content means higher shear modulus; after this time, however, and coinciding with the second calorimetry peak, the 5% FLS sample exhibits the steepest increase and shows the highest values until the end of the measurement.

**Figure 5:**
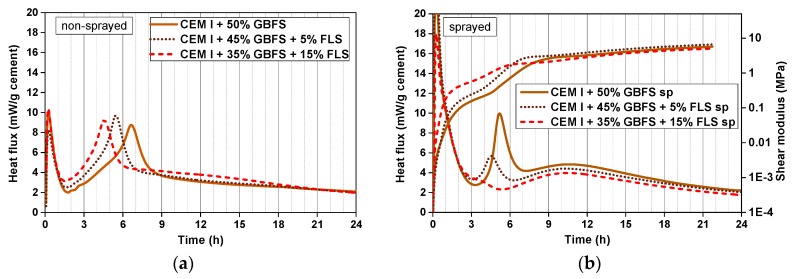
(**a**) Calorimetry curves of non-sprayed samples of mixes five, six, and seven (Table 3 and Table 4), with CEM I + GBFS + FLS; (**b**) calorimetry and shear modulus curves of sprayed samples of mixes five, six, and seven (Table 3 and Table 4), with CEM I + GBFS + FLS.

In the samples with GBFS similar trends are observed in non-sprayed systems (Figure 5a): The second peak is shifted to the left with increasing FLS % (1–2 h) and its maximum is reached almost at the same time as in the CEM I systems without slag. In the GBFS systems, however, the peaks are slightly narrower, and the maximum values slightly higher. As in the CEM I systems, the third maximum is more pronounced in the 15% FLS sample.

In the slag-containing sprayed systems (Figure 5b), all with the same cement content, constant w/c and acc/c ratios, (0.8–1.0% SP and 0.6% retarder, referred to cement weight), the second hydration peak reaction starts considerably earlier than in the non-sprayed and the slag-free systems (despite the SP content being similar, and the retarder content being slightly higher, 0.6%, both referred to the cement weight). The height of the second peak decreases considerably with FLS content and it is almost invisible for the 15% FLS sample, where the end of the first peak and the beginning of the second cannot be distinguished. The shear modulus increases proportionally to the FLS content up to the 5^th^ h where, similarly to the slag free systems, the 5% FLS sample takes over, reaching higher values up to the end of the test. The third hydration maximum, similarly to the second peak, decreases with the FLS content.

**Figure 6:**
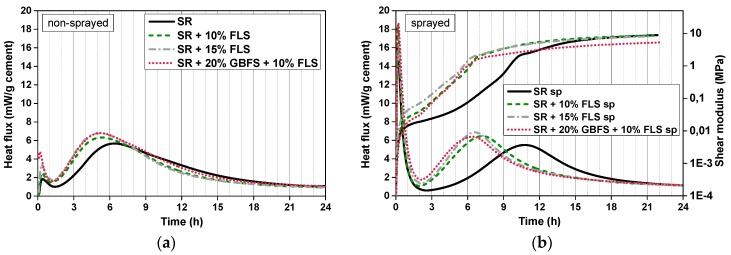
(**a**) Calorimetry curves of non-sprayed samples of mixes 9, 10, 11, and 12 (Table 3 and Table 4), with CEM SR + GBFS + FLS; (**b**) calorimetry and shear modulus curves of sprayed samples of mixes 9, 10, 11, and 12 (Table 3 and Table 4), with CEM SR + GBFS + FLS.

In the non-sprayed CEM SR systems shifts of the main hydration peak are observed: The replacement of cement by 10–15% FLS promotes the earlier onset of the reaction (~2 h shift). The height of the peak also increases with the replacement content (Figure 6a). The differences between systems with 15% FLS and 20% GBFS + 10% FLS are negligible in this region. As opposed to CEM I systems, no third maximum is observed in CEM SR samples.

In the sprayed systems (with accelerator) the first peak is much higher than in non-sprayed (without accelerator). The induction period in FLS-free samples is longer than in the non-sprayed systems, similarly to CEM I systems. In this case, however, the effect of the superplasticizer (0.3–0.4%) and retarder (0.5–0.6%, referred to cement weight) has not completely been removed by the accelerator (despite the retarder content being similar and the SP content being considerably lower than in CEM I systems): The main hydration reactions take place 4–5 h later in sprayed than in non-sprayed systems. The main peak shift to the left is very pronounced from 0% to 10% FLS (~4.5 h) in the sprayed samples. However, further replacement by FLS (and GBFS) leads to only minor shifts. The acceleration effect due to the filler is much more pronounced in sprayed than in non-sprayed systems: The major slope increase of the shear modulus takes place 5 h earlier in FLS containing samples than in the non-substituted CEM SR sample. However, after the main hydration peak, the shear modulus of the sample with no cement replacement continues increasing at a higher rate and it reaches similar end values as the FLS samples.

### 3.2. XRD

**Figure A2:**
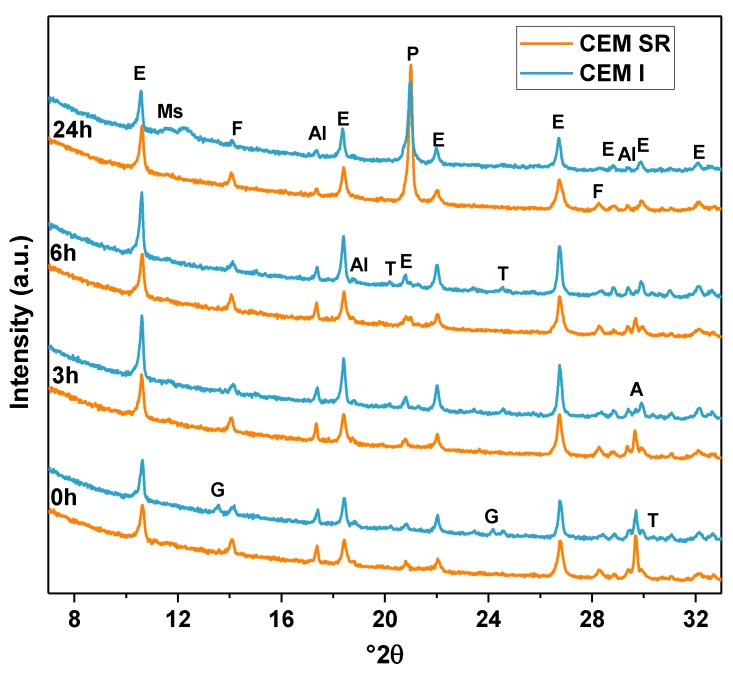
XRD patterns of CEM I and CEM SR sprayed systems with no aggregates after zero, 3, 6, and 24 h. E: Ettringite, Ms: Monosulfoaluminate, Al: Alite, T: Tricalcium aluminate, G: Gypsum, F: Ferrite, P: Portlandite.

**Figure A3:**
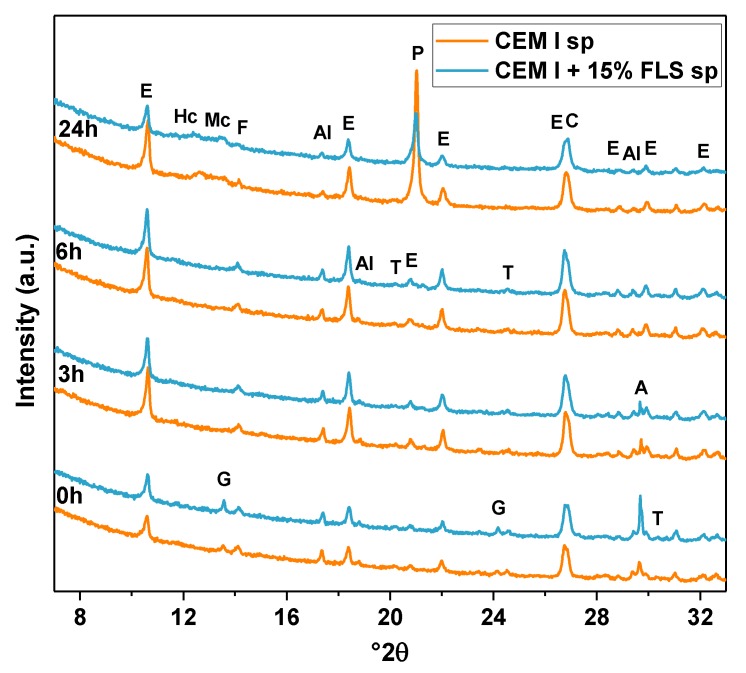
XRD patterns of mixes two and four (Table 3 and Table 4), with CEM I and CEM I + 15% FLS, respectively, after zero, 3, 6, and 24 h. E: Ettringite, Hc: Hemicarboaluminate, Mc: Monocarboaluminate, Al: Alite, T: Tricalcium aluminate, C: Calcite, G: Gypsum, F: Ferrite, P: Portlandite.

**Figure A4:**
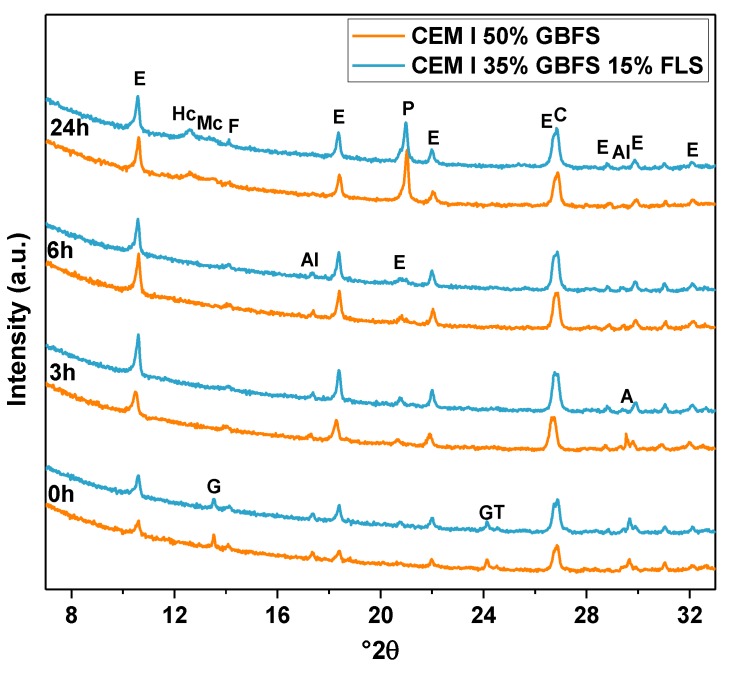
XRD patterns of GBFS sprayed systems with and without FLS after zero, 3, 6, and 24 h. E: Ettringite, Hc: Hemicarboaluminate, Mc: Monocarboaluminate, Al: Alite, T: Tricalcium aluminate, C: Calcite, G: Gypsum, F: Ferrite, P: Portlandite.

The hydration of the two types of systems, CEM I and CEM SR, produces a different mineralogy: In CEM SR samples ettringite increases with time up to the 24^th^ h and no conversion to AFm is observed (XRD patterns shown in the Appendix A, Figure A2). In the CEM I system formation of monosulfoaluminate (in the absence of limestone), coupled with a reduction of the ettringite content, is visible after 24 h (XRD patterns shown in the Appendix A, Figure A2). Just after spraying gypsum forms in the CEM I systems, not visible again after 3 h; the anhydrite present in these (CEM I) systems is more rapidly consumed, disappearing after 6 h, when C_3_A is still available. In the CEM SR system anhydrite remains quite high up to the 6^th^ h whereas C_3_A cannot be detected already after spraying. The portlandite peaks intensity after 24 h is higher in the CEM SR systems. No significant differences are encountered in the mineralogy of the CEM I systems with and without FLS after zero, 3, 6, and 24 h (XRD patterns shown in the Appendix A, Figure A3). In both cases ettringite peaks grow up to the 6^th^ h and then decrease coinciding with the appearance of Hc and Mc. The reduction in the ettringite peak seems more pronounced in the sample with FLS, where more Mc and less Hc form. The portlandite peak, only clearly visible after 24 h, is higher in the sample without FLS. However, this sample also contains more cement, and as amorphous phases are forming, the relative intensity of the peaks cannot be directly used to compare quantities of phases formed. The patterns of GBFS samples (included in the Appendix A, Figure A4) show smaller XRD peaks for all phases than the GBFS-free samples. The reduction of the ettringite peak after 24 h is much less pronounced than in the GBFS-free samples, and the anhydrite is consumed in this case earlier in the samples with FLS.

**Figure 7:**
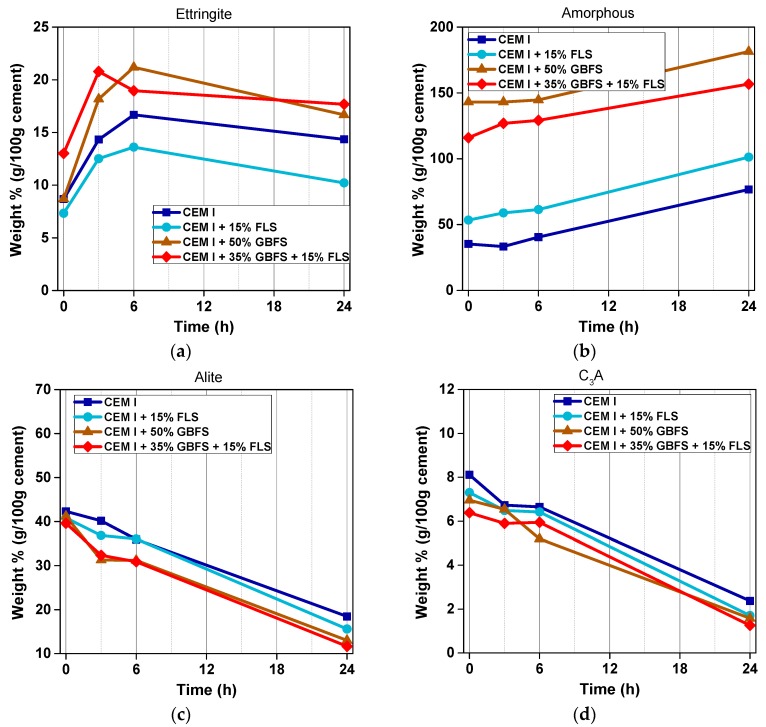
Quantities of ettringite (**a**), amorphous content (**b**), alite (**c**) and C_3_A (**d**), expressed in weight % (g/100 g cement), as determined by Rietveld analysis, in mixes two, four, five, and seven (Table 3 and Table 4), with CEM I and CEM I + 15% FLS, CEM I + 50% GBFS, and CEM I + 35% GBFS + 15% FLS, respectively. Please note that because GBFS samples contain 50 wt.% cement and 35–50% amorphous GBFS, the amorphous content in these samples can be higher than 100%: 100 g of amorphous material per 100 g of cement in the sample are equivalent to 50 g of amorphous materials per 100 g of sample (considering the GBFS completely amorphous).

The quantities of ettringite formed in CEM I and (CEM I + GBFS) systems just after spraying varied between 7% and 13% (referred to cement mass), the highest content corresponding to the sample with 35% GBFS and 15% FLS (Figure 7a), which also shows the lowest initial C_3_A content (Figure 7d). Despite similar cement content, w/c and ac/c ratios in both GBFS systems, the samples with FLS form more ettringite, in accordance with the increase in shear modulus. Samples without slag formed less ettringite than those with slag, with a higher acc/c ratio. In all cases the amounts of ettringite formed after spraying were higher than those calculated from the sulfate and aluminum present in the accelerator (between 5 and 9.5 g per 100 g cement), the difference between both values being especially marked for the 35% GBFS sample. Initial C_3_A is higher in the samples with no limestone (and in those with no slag): At early stages C_3_A dissolved faster in the presence of calcite. Remarkable is the reduction of C_3_A from 3 to 6 h in the 50% slag sample, corresponding to the very pronounced (second) peak in the calorimetry curves (Figure 5b).

The presence of limestone (together with the different w/c and ac/c) led to a higher amorphous content at all times in the slag free samples. In the GBFS samples, the amorphous content (per gram of cement) includes that of the GBFS and is thus not ideal for comparison (Figure 7b). Nevertheless, subtracting the corresponding content of the GBFS (assuming it has not reacted), the higher amorphous content in the slag samples can be deduced. The amorphous content in limestone-containing samples increases from zero to 3 h more pronouncedly than in limestone-free samples. From 3 to 6, the increase in both amorphous and ettringite content is more pronounced for the limestone-free samples. The higher transformation of ettringite into AFm phases in the 50% GBFS sample also translates in a steeper increase in the amorphous content from 6 to 24 h, as compared to the 35% GBFS sample. Alite values show a faster dissolution in slag samples, especially pronounced from zero to 3 h (Figure 7c). Differences in alite content due to the presence of limestone are minor in slag-containing samples; in slag-free systems, the presence of limestone leads to higher dissolution rates of alite after 3 and 24 h. The effect of FLS was also noticeable in the unit cell of the ettringite formed: FLS containing samples produced bigger unit cells than those without limestone (values included in the Appendix A, Table A1). Additionally, ferrite was found to dissolve faster in FLS samples in both systems, with and without slag, especially in the first hour.

### 3.3. FTIR

**Figure 8:**
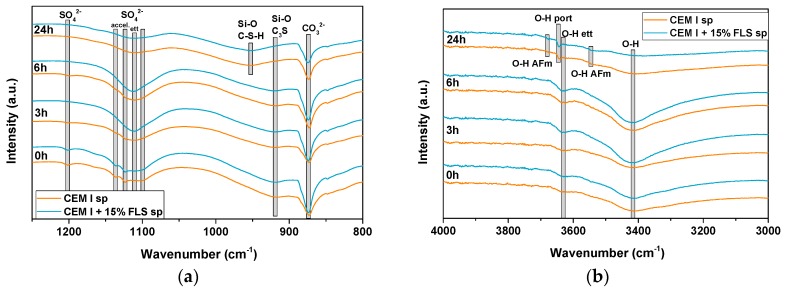
FTIR spectra of mixes two and four (Table 3 and Table 4), with CEM I and CEM I + 15% FLS, respectively, after zero, 3, 6, and 24 h, wavenumber 1250–800 cm^−1^ (**a**) and 4000–3000 cm^−1^ (**b**).

The faster progression of the early hydration reactions in lab-sprayed FLS systems was also confirmed by FTIR spectroscopy (Figure 8). The disappearance of the bands in the region ~1100 and ~1200 cm^−1^, corresponding to the S–O stretching vibration [46] of sulfate-containing phases at early stages progresses much faster in the FLS (CEM I) sample, where after 3 h only the band at ~1111 cm^−1^, corresponding to ettringite, can be seen. In the FLS-free (CEM I) sample the other bands (1100, 1123, 1136, and 1200 cm^−1^) remain after 6 h (together with that of ettringite). Additionally, the consumption of carbonate to form AFm phases (indicated in the XRD patterns) is appreciated in the significant reduction with time of the C–O band at ~875 cm^−1^ [47] in the FLS samples. The corresponding band in the FLS-free systems remains almost constant over time. The shift of the Si–O absorption band, from ~918 cm^−1^ (0–6 h) to ~953 cm^−1^ (24 h), corresponding to formation of C–S–H from the hydration of alite [48], does not present significant differences between the two types of samples. The O–H stretching bands corresponding to ettringite (3415 and 3625 cm^−1^) [48] increase in both cases up to 6 h and then decrease. The one corresponding to portlandite, at ~3644 cm^−1^ [49,50], is only clearly visible after 24 h in both systems. Overlapping of bands associated to different AFm phases and also ettringite make the clear differentiation and identification of aluminate reaction products difficult. However, it can be clearly distinguished that bands at ~3543 and 3675 cm^−1^, attributed to AFm phases [51], are more prominent in the FLS samples.

### 3.4. TGA

**Figure 9:**
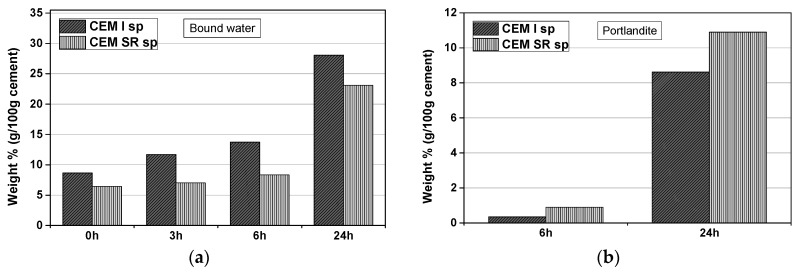
(**a**) Bound water in the samples from mixes one and eight (Table 3 and Table 4), after zero, 3, 6, and 24 h (CEM I and CEM SR sprayed samples with no aggregates), and (**b**) portlandite content in the samples after 6 and 24 h, both expressed as wt.% (g per 100 g cement), as deduced from TG measurements.

CEM I systems combine water faster and to a greater extent than those with CEM SR cement, the increase in bound water being specially pronounced up to 3 h (Figure 9a), where according to the XRD results most of ettringite forms. The amount of portlandite in CEM SR systems after 24 h is higher than in CEM I ones (Figure 9b).

**Figure 10:**
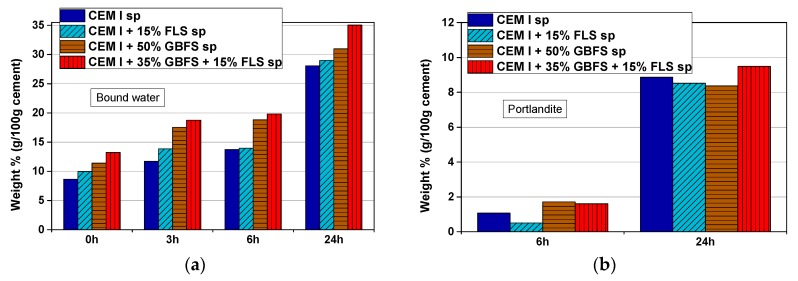
(**a**) Bound water after zero, 3, 6, and 24 h in samples from mixes two, four, five, and seven from Table 3 and Table 4 (CEM I + FLS + GBFS sprayed samples), and (**b**) portlandite content in the samples after 6 and 24 h, both expressed in wt.% (g/100 g cement), as deduced from TG measurements.

The replacement of cement in CEM I and slag in (CEM I + GBFS) systems by FLS leads to an increase in the amount of bound water at all ages analyzed (Figure 10a). The portlandite content is 0–2% after 6 h and 8–9.5% after 24 h in all samples (Figure 10b).

**Figure 11:**
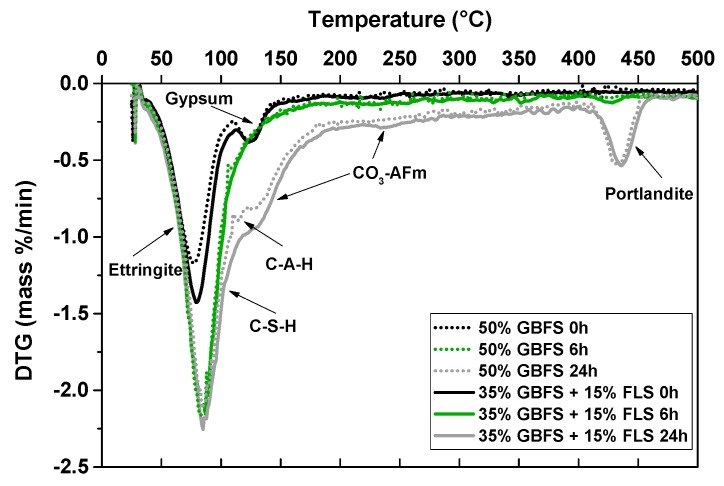
DTG curve of samples from mixes five and seven (Table 3 and Table 4) after zero, 6, and 24 h.

The bound water includes contributions from ettringite, AFm, calcium aluminate hydrates, C–S–H, portlandite, and gypsum, if present, as observed by XRD, FTIR, and TGA. As an example, the derivative curves of the TG graphs (DTG) of mix five (CEM I + 50% GBFS) and mix seven (CEM I + 35% GBFS + 15% FLS) samples, after zero, 6 and 24 h, are shown in Figure 11. After zero h the water corresponds to the dehydration of ettringite and gypsum (see the second peak around 125 °C in Figure 11), as confirmed by XRD. After 3 and 6 h the water comes from ettringite, calcium aluminate hydrates, and to a smaller extent from C–S–H, indicated by the broadening of the first decomposition peak and its shift to higher temperatures. Very small amounts of portlandite are formed after 6 h (see small peak at ~425 °C). In addition, it is worth remarking the very small peak in FLS-free samples at ~110 °C, both after 6 and 24 h (and absent after 3 h), corresponding to aluminate hydration products (C–A–H in the graph). After 24 h, apart from the already mentioned phases, there is an extra contribution from CO_3_-AFm phases: See shoulder around 135 °C and less pronounced hump at ~230 °C, both more important in FLS-containing samples. The DTG curves of the other CEM I samples included in Figure 10 show similar features

### 3.5. SEM

**Figure 12:**
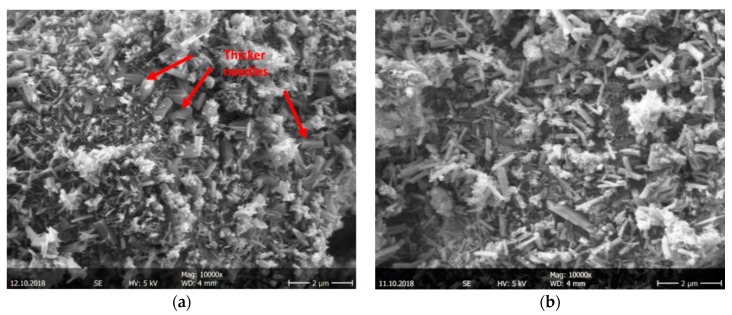
SEM image of mix eight (Table 3) with CEM SR (**a**), and mix one (Table 3) with CEM I (**b**), after 6 h hydration.

The ettringite needles formed in CEM SR samples are shorter and thicker than those in CEM I samples. Figure 12a,b show representative areas of both systems after 6 h of hydration (see arrows pointing at short and thick needles in CEM SR sample in Figure 12a). The longest needles reach ~0.7 and 1.0 μm in length in CEM SR and CEM I samples, respectively, and there are more ‘long’ needles in the CEM I than in the CEM SR matrix.

**Figure 13:**
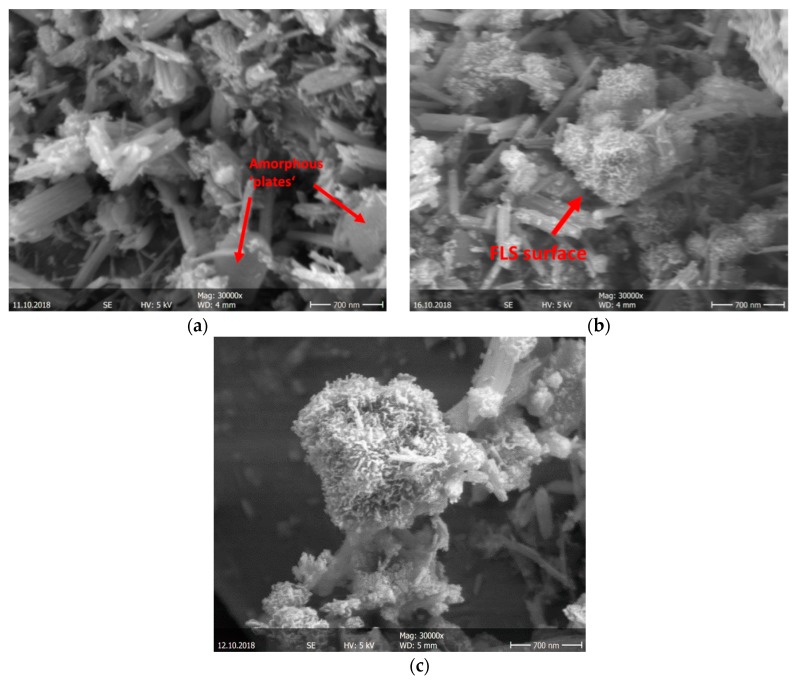
SEM images of (**a**) mix two (Table 3), CEM I without FLS; (**b**) mix four (Table 3), CEM I with 15% FLS; (**c**) mix seven (Table 3), CEM I mix with GBFS and FLS, after 6 h hydration.

Differences in the morphology of CEM I samples with FLS (Figure 13b) and without FLS (Figure 13a) are visible at high magnification (Figure 13) after 6 h: Small round objects (~0.5–1.0 μm) with a reacted surface are identified within the matrix of the FLS samples both with and without GBFS (Figure 13b,c respectively). These little ‘balls’ are identified as the FLS grains and the ‘sponge-like’ material growing on their surface as hydration products. Amorphous ‘plates’, corresponding to aluminate hydrates, are noticeable especially in the FLS-free sample (see arrows in Figure 13a).

### 3.6. Real Scale Early Strength

**Figure 14:**
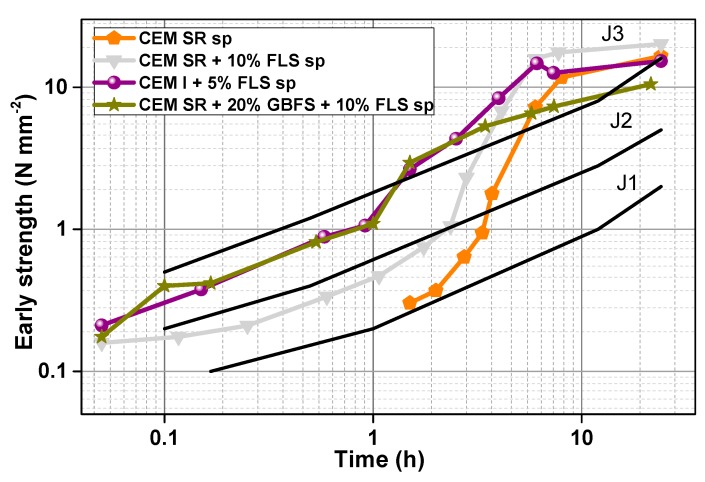
Early strength of real scale mixes three, nine, 10, and 12 (Table 5). The three strength classes, J1, J2 and J3, are those established by the Austrian Guidelines for Sprayed Concrete [45].

The effect of FLS addition on strength development was validated in real scale tests. 10% FLS in CEM SR systems led to a significant increase in compressive strength throughout the first day (Figure 14), similarly to what happened in the lab-sprayed mixes (Figure 6b). In this case, as opposed to the lab-scale test, the w/c (0.51) was kept constant, as well as the ac/c ratio (0.08) (Table 5). Replacement of CEM SR cement by 20% GBFS in samples with 10% FLS also lead to a further improvement of the early strength; this enhancement, however, was only observable in the first 4 h, after which the GBFS-containing sample increased strength at a slower pace than the GBFS-free sample. It is worth remarking that the (FLS-free) CEM SR sample only reached ‘measurable’ strength after the first 1.5 h, this being the reason why no data points are shown before that time. The mix with CEM I cement and 5% FLS replacement, also with 0.51 w/c ratio and 0.086 ac/c ratio, showed very high early strength values. During the first 3–4 h, the strength development of samples CEM I + 5% FLS and CEM SR + GBFS + FLS is very similar. However, after 4 h, the CEM I + 5% FLS sample continues growing at a higher rate, reaching higher values after 24 h. The compressive strength of the shotcrete mixes after 28 and 90 days, measured according to [45], is given in the Appendix A (Table A2).

## 4. Discussion

The addition of fine limestone fillers has a direct impact on the hydration progress of (aluminum sulfate) accelerated concrete mixes. The deconvolution of the various effects of these fillers is more complex than in the case of non-accelerated mixes, where the so-called filler effect mainly applies to C–S–H formation. In accelerated concrete, early formation of significant amounts of sulfoaluminate hydrates conditions the further progression of the hydration reactions:

(1) The first aluminate reaction, controlled by the accelerator and cement composition, and with ettringite as main hydration product, is enhanced by the fine limestone filler, probably due to the supply of extra nucleation sites and, to a minor extent, to the extra calcium available from the dissolution of limestone.
(2) The sulfate depletion is controlled by the formation of ettringite: Enhancement of (first) aluminate reaction in the presence of FLS accelerates the sulfate depletion. However, because of the lower amounts of aluminate available to form AFm and/or C–A–H phases, the earlier (second) aluminate reaction is also less pronounced in the presence of limestone, as indicated by the calorimetry curves of GBFS samples (Figure 5b). The effect of extra nucleation sites provision by FLS for calcium aluminate hydrates other than ettringite [52] seems to be counterbalanced and surpassed by the reduction in available aluminate. At the very early stages of hydration, the low pH of the accelerator (~3) may promote limestone dissolution [9], with the consequent supply of extra carbonate ions, enhancing its reactive role in the formation of carbonate-AFm phases. Both AFm and C-A-H phases formed (Figure 8 and Figure 13) at early hydration times show amorphous nature and only after 6 h of hydration slightly crystalline CO_3_-AFm phases start to become ‘visible’ with XRD. The composition of these early aluminate hydrates is expected to be a mix of SO_3_-, CO_3_-, OH-AFm, and/or C_3_AH_6_.
(3) Finally, the silicate reaction is influenced by all the previous reactions, by ettringite, AFm and aluminate hydrates formation, and also by the presence of limestone. Similarly to non-accelerated concrete, limestone filler promotes the formation of calcium silicate hydrates. Additionally, in accelerated systems, the extra consumption of calcium during the enhanced formation of ettringite promotes C_3_A and alite dissolution, as shown in Figure 7d. On the other side, the hindering of alite dissolution due to the high aluminum concentration in solution [53] and the partial inhibition of calcium silicate hydrate formation because of the space restrictions created from the aluminate hydrates formation [54], will also be affected by the presence of limestone. Earlier sulfate depletion and aluminum consumption, together with the less pronounced aluminate reaction, help reduce the partial repression of the silicate reaction. The increase of the ettringite unit cell in FLS samples (with and without slag) is associated with an enhanced dissolution of ferrite and the possible substitution of iron by aluminum in the ettringite structure [55].

Mixes used for sprayed concrete applications have special performance requirements: They need to provide very high early strength in the very first minutes-hours of hydration. To achieve this, the whole system needs to be optimized: Cement, SCMs, accelerator, mix proportioning, other chemical admixtures, etc. In most cases, the couple cement-accelerator is considered as a unit that needs to work well together: The accelerator has to be adequate for the cement, and vice versa. In the data presented, a special cement used for sprayed concrete applications and a standard cement used for sulfate resistant concrete application were tested in combination with an aluminum sulfate based accelerator (with Al/S molar ratio ~0.7). The main difference between the two cements lies in the C_3_A, responsible for the supply of calcium (and aluminum) for the early aluminate reaction and formation of ettringite. In the high C_3_A cement, the greater formation of ettringite allows for a faster initial hardening of the shotcrete. Additionally, the high C_3_A and the consequent early (second) aluminate reaction (after the sulfate depletion) allows for an important increase in strength around the 6^th^ h. This early sulfate depletion, usually not desired in non-accelerated systems, is key for the strength development of these accelerated systems. If properly optimized, its impact on the partial inhibition of the further silicate reaction should be minor and not as important as the beneficial effect during the first hours. The high reactivity of this C_3_A-rich cement is not only due to the amount of C_3_A but also to the ratio cubic/orthorhombic C_3_A (0.7%/10.8%), controlled by the alkali content in the raw mix and the cooling rate [56]. In the presence of sulfate the orthorhombic C_3_A is more reactive than the cubic polymorph [57]. The low C_3_A cement, on the other side, forms smaller amounts of ettringite but, more important than that, it also increases strength at a slower pace. Despite the silicate reaction being more pronounced in these low C_3_A systems, and actually forming more C–S–H (and portlandite) after 24 h, the gap between ettringite and silicate formation, where the strength barely increases, may make this cement not suitable for shotcrete applications. Contrary to what was stated by Salvador et al. [9] the better sulfation of low C_3_A cements does not necessarily give better results in terms of strength development for shotcrete products. Formation of AFm phases is avoided and silicate reaction is thus not inhibited. However, during the crucial time between 3 and 6 h an extra push in strength is needed to reach the requirements for shotcrete early strength development. Despite the low C_3_A cement being better sulfated, its use in combination with a suspension accelerator with Al/S molar ratio of ~0.7 is not adequate for sprayed concrete. The presence of limestone in the low C_3_A cement systems makes them suitable for shotcrete applications due to the enhancement of both the ettringite and silicate formation. Additions of 10% FLS increased the compressive strength of the mixes at all times during the first day, producing strength evolution comparable to high C_3_A cement mixes. An optimal combination of accelerator dosage, dependent in turn on the accelerator composition, and limestone addition would allow for the ‘perfect’ shotcrete mix, where ettringite formation is enhanced, the aluminate reaction partly inhibited and the silicate reaction if not promoted, at least only inhibited to a minor extent. In the mixes studied here the beneficial impact of FLS additions is appreciated in all cases: Higher FLS content, either replacing cement or added to the mix, leads to greater early hydration and strength development. In the slag systems, where FLS is replacing slag and the cement content is kept constant, the accelerator dosage, 6.0% referred to the binder and 11.6% referred to the cement, produces large amounts of ettringite and probably C–A–H/AFm phases in the first hour. The addition of limestone further promotes these early reactions, accelerating the sulfate depletion and reducing the (second) aluminate reaction, this all contributing to an increase in the strength up to the 6^th^ h, progressively with the FLS content. After that, however, the silicate reaction is partly inhibited by the previous reactions, and an optimum in strength development is found for 5% FLS. The high shear modulus and strength values reached by GBFS samples, despite the lower cement content, may as well be an indication of the early hydration of slag in accelerated systems, as it was recently reported [58].

The results presented here are very relevant for the shotcrete industry as they prove that fine limestone can help compensating the early strength losses resulting from the substitution of cement clinker by SCMs. In applications such as tunneling, where the shotcrete is responsible for the stabilization of the rock ground and the maintenance of its own load capacity, early strength of shotcrete cannot be compromised. Up to now, very rich cement mixes (in combination with setting accelerators) have been used to reach the strength required. However, in order to contribute to the reduction of cement CO_2_ emissions and to improve the durability properties of shotcrete, the cement content in the future shotcrete mixes should be further reduced [59]. The results presented here show that these mixes are feasible if the cement is replaced by SCMs, such as slag and/or fly ash, and very fine limestone fillers. Additionally, the use of fine limestone should allow for an optimization of the setting accelerator dosage without compromising early strength. The implications of these limestone effects on early hydration should also be taken into account in mix designs including calcite aggregates, where the finest fraction should act in a way similar to the fine limestone powder used in this study.

## 5. Conclusions

The “filler effect” of fine limestone in non-accelerated and accelerated mixes has been compared. Additionally, the filler effect in shotcrete has been validated in real scale spraying tests. From the results obtained the following conclusions have been drawn:

- Fine limestone enhances early hydration reactions, including ettringite, and in some cases calcium aluminate hydrates, formation. This enhancement conditions the further development of the silicate and aluminate reactions.
- In high C_3_A cement systems, the presence of fine limestone also translates in most cases in an earlier sulfate depletion and subsequent aluminate reaction. 5% FLS seems to be an adequate optimum in terms of strength development during the first 24 h in the samples studied.
- In mixes with low C_3_A cement, the impact of the filler on the silicate reaction is considerably more important than in those with high C_3_A cement, making the systems even comparable in terms of strength to special shotcrete binders.
- There is potential for the optimization of the accelerator/limestone ratio for the various cements: Lower amounts of accelerator will be needed to achieve the same early strength in the presence of fine limestone.
- The increase in early strength produced by the fine limestone will help reduce the cement clinker content in shotcrete mixes without compromising, or while even improving, the early mechanical properties.
- Future work on the hydration mechanisms involved in the filler effect of shotcrete should focus on pore solution analysis, the use of other fillers than limestone, and characterization of the pore structure development. Additionally, durability investigations are needed to clarify the various effects of limestone addition, such as the formation of CO_3_-AFm.

## Figures and Tables

**Table 1:** Composition of cements used in lab experiments, determined by XRD + Rietveld analysis.

Phase (wt.%)	CEM I	CEM SR
Alite	55.0	58.2
Belite	13.1	12.6
Aluminate cubic	0.7	0.5
Aluminate ortho	10.8	1.2
Ferrite	7.4	12.3
Periclase	4.2	-
Anhydrite	3.8	3.4
Arcanite	2.0	0.4
Bassanite	1.7	0.5
Calcite	0.9	9.5
Portlandite	0.3	0.9
Aphthitalite	-	0.5
wRp	5.4	4.6

**Table 2:** Oxide composition of cements, granulated blast furnace slag (GBFS), fine limestone (FLS) and aggregates used for the lab experiments, as determined by X-ray fluorescence analysis.

Oxide (wt.%)	CEM I	CEM SR	GBFS	FLS	Aggregates (Nekafill)
Na_2_O	0.5	0.4	0.4	0.1	-
MgO	4.0	1.2	8.7	1.5	2.6
Al_2_O_3_	5.4	2.9	11.9	0.1	0.4
SiO_2_	20.0	20.2	39.5	1.1	1.0
P_2_O_5_	0.1	0.1	-	-	-
SO_3_	3.0	2.3	1.7	-	-
K_2_O	1.0	0.4	1.0	-	-
CaO	61.5	64.0	34.6	54.6	51.6
TiO_2_	0.2	0.2	0.5	-	-
MnO	0.1	0.1	1.4	-	-
Fe_2_O_3_	2.8	4.3	0.4	0.1	0.1
LOI	1.3	4.0	-	42.6	44.3

**Table 3:** Summary of mixes investigated: Binder composition (mass percentages).

Mix	CEM I	CEM SR	FLS	GBFS	Aggregates (Nekafill)	Real Scale
1	100%					
2	100%				X	
3	95%		5%		X	X
4	85%		15%		X	
5	50%			50%	X	
6	50%		5%	45%	X	
7	50%		15%	35%	X	
8		100%				
9		100%			X	X
10		90%	10%		X	X
11		85%	15%		X	
12		70%	10%	20%	X	X

**Table 4:** Composition of the mixes sprayed in the lab (MiniShot). Weight percentages of superplasticizer (SP), retarder (Retar), and accelerator (Accel) are referred to the binder (cement + GBFS + FLS).

Mix	Cement (kg/m^3^)		FLS (kg/m^3^)	GBFS (kg/m^3^)	Nekafill (kg/m^3^)	Water (kg/m^3^)	SP (wt.%)	Retar (wt.%)	Accel (wt.%)	w/b
1	CEM I	1262	-	-	-	593	0.7	0.5	6.0	0.47
2		1097	-	-	352	516	1.0	0.5	6.0	0.47
3		1039	55	-	352	514	1.0	0.5	6.0	0.47
4		924	163	-	352	511	1.2	0.4	6.0	0.47
5		557	-	521	362	507	0.5	0.3	6.0	0.47
6		558	49	470	363	506	0.4	0.3	6.0	0.47
7		560	146	366	365	504	0.5	0.3	6.0	0.47
8	CEM SR	1329	-	-	-	585	0.3	0.5	6.0	0.44
9		1155	-	-	353	508	0.4	0.5	6.0	0.44
10		1032	115	-	353	504	0.3	0.5	6.0	0.44
11		971	171	-	353	503	0.3	0.4	6.0	0.44
12		796	114	227	353	500	0.3	0.4	6.0	0.44

**Table 5:** Mixes sprayed in real scale tests. Weight percentages of superplasticizer (SP) and accelerator (Accel) are referred to the binder (cement + GBFS + FLS).

Mix	Cement (kg/m^3^)		FLS (kg/m^3^)	GBFS (kg/m^3^)	Aggregates (kg/m^3^)	Water (kg/m^3^)	SP (wt.%)	Accel (wt.%)	w/b
3	CEM I	391	20	-	1852	200	1.0	8.2	0.49
9	CEM SR	406	-	-	1888	206	1.0	8.0	0.51
10		362	40	-	1900	184	1.0	7.3	0.46
12		281	40	80	1885	188	1.0	7.0–8.0^1^	0.47

^1^ In sample 12 the exact amount of accelerator used could not be measured due to technical difficulties. The 7.0%–8.0% corresponds to an estimation based on the planned values and the experience gained from the previous tests.

## Appendix A

**Table A1:** Ettringite unit cell ‘a’ in mixes with CEM I, CEM I + 15% FLS, CEM I + 50% GBFS, and CEM I + 35% GBFS + 15% FLS after zero, 3, 6, and 24 h.

Time (h)	CEM I	CEM I + 15% FLS	CEM I + 50% GBFS	CEM I + 35% GBFS + 15% FLS
	Ettringite Unit Cell a (Å)			
0	11.2079	11.2145	11.2074	11.2114
3	11.2123	11.2122	11.2122	11.2136
6	11.2092	11.2124	11.2119	11.2123
24	11.2089	11.2137	11.2097	11.2121

**Table A2:** Compressive strength measured on shotcrete drilled cores (100 mm diameter × 100 mm height) after 28 and 90 days, according to [45] (average values from 3–4 drilled cores).

Time (days)	CEM SR sp	CEM SR + 10% FLS sp	CEM I + 5% FLS sp	CEM SR + 20% GBFS + 10% FLS sp
	Compressive Strength (MPa)			
28	55.6	59.9	41.1	52.4
90	67.5	70.2	59.0	64.7
